# Sensitivity of screening mammography by density and texture: a cohort study from a population-based screening program in Denmark

**DOI:** 10.1186/s13058-019-1203-3

**Published:** 2019-10-17

**Authors:** My von Euler-Chelpin, Martin Lillholm, Ilse Vejborg, Mads Nielsen, Elsebeth Lynge

**Affiliations:** 1 Department of Public Health, University of Copenhagen, Øster Farimagsgade 5, DK-1014 Copenhagen K, Denmark; 2 Biomediq, Fruebjergvej 3, DK-2100 Copenhagen Ø, Denmark; 3 Department of Computer Sciences, Universitetsparken 5, University of Copenhagen, DK-2100 Copenhagen Ø, Denmark; 4 Department of Radiology, Centre of Diagnostic Imaging, University Hospital Copenhagen Rigshospitalet, Blegdamsvej 9, DK-2100 Copenhagen Ø, Denmark; 5 Nykøbing Falster Hospital, University of Copenhagen, Ejegodsvej 63, DK-4800 Nykøbing Falster, Denmark

**Keywords:** Mammography, Mammographic density, BI-RADS, Parenchymal texture marker, Sensitivity

## Abstract

**Background:**

Screening mammography works better in fatty than in dense breast tissue. Computerized assessment of parenchymal texture is a non-subjective method to obtain a refined description of breast tissue, potentially valuable in addition to breast density scoring for the identification of women in need of supplementary imaging. We studied the sensitivity of screening mammography by a combination of radiologist-assessed Breast Imaging Reporting and Data System (BI-RADS) density score and computer-assessed parenchymal texture marker, mammography texture resemblance (MTR), in a population-based screening program.

**Methods:**

Breast density was coded according to the fourth edition of the BI-RADS density code, and MTR marker was divided into quartiles from 1 to 4. Screening data were followed up for the identification of screen-detected and interval cancers. We calculated sensitivity and specificity with 95% confidence intervals (CI) by BI-RADS density score, MTR marker, and combination hereof.

**Results:**

Density and texture were strongly correlated, but the combination led to the identification of subgroups with different sensitivity. Sensitivity was high, about 80%, in women with BI-RADS density score 1 and MTR markers 1 or 2. Sensitivity was low, 67%, in women with BI-RADS density score 2 and MTR marker 4. For women with BI-RADS density scores 3 and 4, the already low sensitivity was further decreased for women with MTR marker 4. Specificity was 97–99% in all subgroups.

**Conclusion:**

Our study showed that women with low density constituted a heterogenous group. Classifying women for extra imaging based on density only might be a too crude approach. Screening sensitivity was systematically high in women with fatty and homogenous breast tissue.

## Background

Randomized controlled trials showed screening mammography to reduce breast cancer mortality by early detection of tumors before they give rise to symptoms [1–4]. The sensitivity of screening mammography depends, however, on the composition of the breast tissue. Mammography works better in fatty than in dense breasts [5]. In 2004, the personal experience of breast cancer detected shortly after a normal mammogram of her dense breast led Nancy Cappello to campaign for women’s right to be informed, if they had a dense breast on their screening mammogram. The first breast density notification law was passed in Connecticut, USA, in 2009, and notification is now a legal requirement in 38 states [6].

The Breast Imaging Reporting and Data System (BI-RADS) forms the basis for the categorization of breast density. The present fifth edition of BI-RADS distinguishes between fatty, scattered, heterogenously dense, and extremely dense breast tissue [7]. Approximately 50% of women undergoing screening in the USA fall into one of the two latter categories [8]. The legislation requires these women to be informed about their dense breast tissue and—varying by state—advised to have supplemental imaging undertaken, but no supplemental imaging modality is yet considered standard of care for women with dense breasts [9].

The BI-RADS density score is assessed by a radiologist, and concern has been raised as to whether the breast density legislation could potentially lead to downgrading of density, to avoid supplementary imaging, or to upgrading of density, to minimize liability, leading to a wish for development of automated breast density evaluation systems [10]. Computerized assessment of the parenchymal texture is a non-subjective method to obtain a more refined description of the breast tissue [11], and it might be a valuable tool to be used in addition to the breast density scoring for identification of women in need of supplementary imaging.

On this basis, we studied the sensitivity of screening mammography by the combination of radiologist-assessed BI-RADS density score and computer-assessed parenchymal texture score. We used data from 55,000 women participating in a population-based screening mammography program in Denmark, where there is no breast density legislation and where only the malignancy score of positive or negative of the screening mammograms forms the basis for the referral of screened women for diagnostic assessment.

## Methods

### Database

The Capital Region of Denmark offers biennial screening to women aged 50–69 years. Women are personally invited to visit one of the five mammography screening clinics in the region. The program uses the Siemens Inspiration digital mammography equipment. At the screening, the radiographer takes a craniocaudal (CC) and a mediolateral oblique (MLO) view. For the present study, we retrieved data on all screening mammography examinations from 1 November 2012 to 31 December 2013. Within the study period, no woman was screened more than once.

All mammography examinations were read and coded independently by two trained radiologists. If the two readers disagreed on the malignancy code, a consensus code was made in a dialog between the two readers, and if necessary, a third independent reader was brought in.

Breast density was coded according to the 2003 fourth edition of the BI-RADS density code [12]. BI-RADS 1 is fatty, where the breast is almost entirely fat (< 25% fibroglandular tissue); BI-RADS 2 is scattered (> 25–50%) fibroglandular; BI-RADS 3 is heterogenously (51–75%) dense; and BI-RADS 4 is dense (> 75%). If the two readers disagreed on the BI-RADS density code, the highest code was used as the consensus code.

The fully automated planimetric mammography texture resemblance marker (MTR) was calculated using a five-layer deep learning texture analysis convolutional neural network (CNN) pipeline by Biomediq [13]. The CNN and thus the MTR marker were trained to separate women with a high risk of breast cancer from women with a low risk of breast cancer, using a case-control subset of the Preventicon Screening Unit in Utrecht, the Netherlands [13]. The training cases included mammograms from 285 women with screen-detected breast cancer and mammograms from 109 women with interval cancers, 384 cases in total. The controls comprised mammograms from 3 age- and acquisition-matched healthy women per case, 1182 in total. For screen-detected cases, the mammogram from the contralateral breast was used for the analysis and for interval cancers the latest available mammogram from the contralateral breast. The laterality of control mammograms was selected to match the case laterality. The MTR marker was trained to separate cases from controls. The images were recorded on a Hologic Selenia FFDM system. We here applied this MTR marker to a new dataset. Methodological and technical details and large-scale validation of the MTR marker can be found in [13, 14], respectively.

The MTR marker for the current study was the average of the MTR scores for the four available images (left/right × CC/MLO). Using the median, minimum or maximum value did not significantly change the results (data not shown). The MTR marker was divided into interquartile ranges, Q1–Q4 for the analysis.

Neither the BI-RADS density score nor the MTR marker was used in the clinical management of women.

The outcome of screening and identification of interval cancer cases was assessed by linkage to the Danish Pathology Register [15]. Linkage between registers was based on the unique personal identification numbers allocated to all persons with a permanent address in Denmark. Pseudo-anonymized linkages were used between Biomediq texture databases and the mammography register.

### Analysis

Women with a positive screening test and breast cancer or ductal carcinoma in situ (DCIS) diagnosed within 6 months of the screening date were defined as screen-detected cancers. Other women were followed up until the next screening date or for 24 months whichever came first, for simplicity called 24 months. Women with a negative screening test and breast cancer/DCIS diagnosed within 24 months after the screening date or with a positive screening test but a negative assessment diagnosed with breast cancer/DCIS within 7–24 months after the screening date were defined as interval cancers, i.e., cancers that were not detected at screening but became symptomatic within 2 years and before the next screen. Women with screen-detected cancers and women with interval cancers together constituted all women with breast cancer. Women with a positive screening test and no diagnosis of breast cancer/DCIS were defined as false positive, and women with a negative screening test and no breast cancer/DCIS were defined as truly negative. The two latter groups together constituted the truly healthy women.

We calculated sensitivity (= screen detected/all women with breast cancer) and specificity (= truly negative on screening /truly healthy) with 95% confidence intervals (CI) by BI-RADS density score, by MTR marker, and by the combination of the two. SAS 9.4, copyright (c) 2002–2012 by SAS Institute Inc., Cary, NC, USA, was used for the analysis.

## Results

**Fig. 1:**
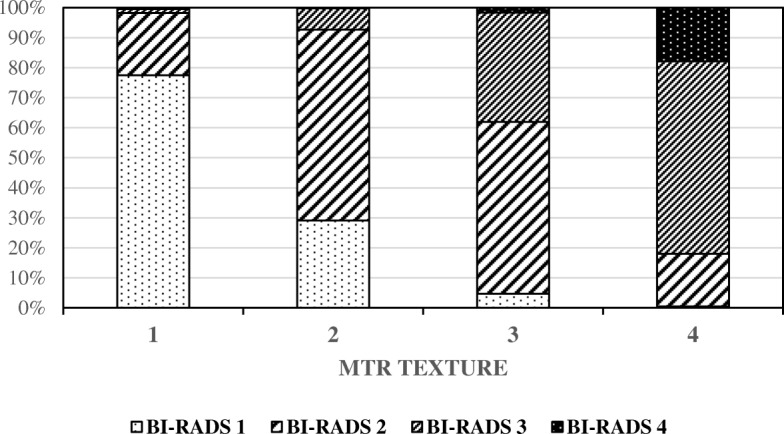
Distribution of the screening population density score (BI-RADS) and texture marker (MTR)

In total, 55,350 women were screened from 1 November 2012 to 31 December 2013. Full data were available for 54,997 women, of whom 28% had BI-RADS density score 1, 40% score 2, 27% score 3, and 5% score 4. The BI-RADS density score and the MTR marker were highly correlated with the majority of women with BI-RADS density score 1 having MTR marker 1 and the majority of women with BI-RADS density score 4 having MTR marker 4, *p* < 0.00001 (Table 1 and Fig. 1).

**Table 1:** Distribution of 54,997 screening examinations by BI-RADS density score by MTR marker, Denmark, 2012–2013

MTR	BI-RADS				Total	Percentage
	1	2	3	4		
1	10,256	3569	217	55	13,652	25
2	3618	7912	2000	122	13,640	25
3	1430	6072	5257	881	13,608	25
4	340	4211	7352	1705	14,097	26
Total	15,644	21,764	14,826	2763	54,997	100
Percentage	28	40	27	5	100	

Chi-square = 43,255.71 with a *p* value < 0.00001 at df = 9

Of the 54,997 women, 53,112 were true negative, 419 true positive, 1304 false positive, and 162 were false negative, out of which 8 were false negative after a positive screening mammogram. The overall sensitivity was 72% (95% CI, 68–76), and the specificity was 98% (95% CI, 97–98). Sensitivity decreased from 78% (95% CI, 69–85) for women with BI-RADS density score 1 to 47% (95% CI, 30–65) for women with BI-RADS density score 4. Specificity varied between 97 and 99% across all density scores. Sensitivity was 76% (95% CI, 67–84) for women with MTR marker 1 and 60% (95% CI, 51–68) for women with MTR marker 4. The range of sensitivity is thus broader by the BI-RADS density score than by the MTR marker. Specificity varied between 97 and 98% (Table 2).

**Table 2:** Distribution of 54,997 screening examinations by sensitivity, specificity, BI-RADS density score, and MTR MEAN score, Denmark 2012–2013

	True positive	False positive	True negative	False negative	Sensitivity, % (95% CI)	Specificity, % (95% CI)
All	419	1304	53,112	162	72 (68–76)	98 (97–98)
BI-RADS 1	88	204	15,327	25	78 (69–85)	99 (98–99)
BI-RADS 2	187	613	20,903	61	75 (70–81)	97 (97–97)
BI-RADS 3	127	420	14,222	57	69 (62–76)	97 (97–97)
BI-RADS 4	17	67	2660	19	47 (30–65)	98 (97–98)
MTR 1	86	242	13,742	27	76 (67–84)	98 (98–98)
MTR 2	111	338	13,166	37	75 (67–82)	98 (97–98)
MTR 3	133	358	13,111	38	78 (71–84)	97 (97–98)
MTR 4	89	366	13,093	60	60 (51–68)	97 (97–98)

**Fig. 2:**
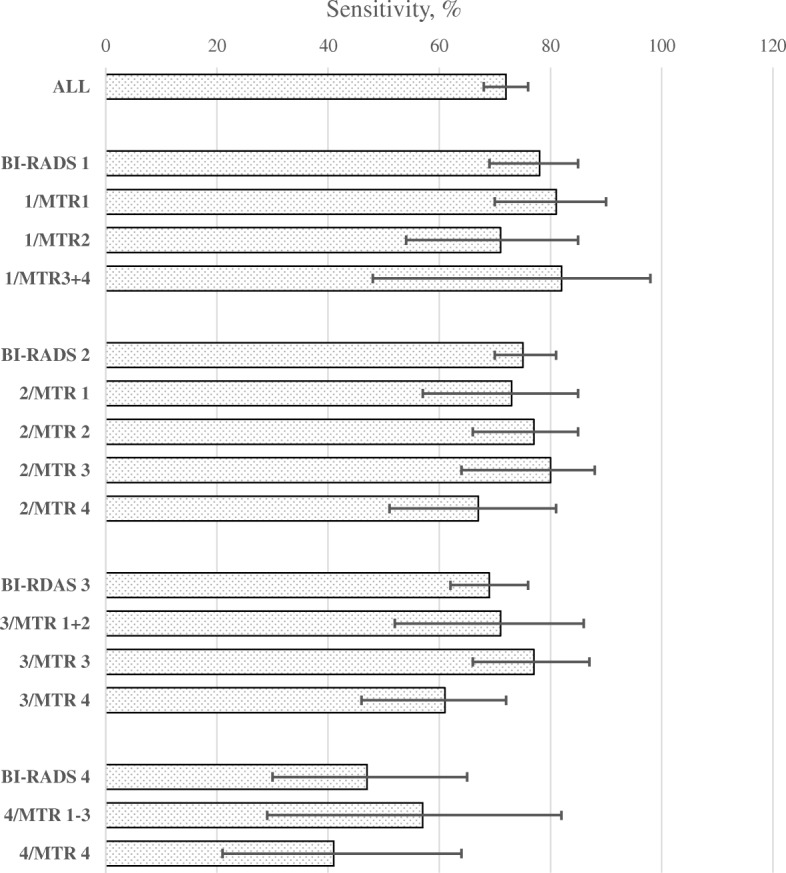
Sensitivity of screening mammography by the combination of density score (BI-RADS) and texture marker (MTR)

Despite the strong correlation between the density score and the texture marker, the combination of the two markers led to the identification of some extra subgroups of women with low screening sensitivity. Screening in women with BI-RADS density score 2 and MTR marker 4 had a sensitivity of only 67% (95% CI, 51–81). In women with BI-RADS density scores 3 and 4, a large proportion of the interval cancers had MTR marker 4, and for women with these combinations, the test had a sensitivity of only 61% (95% CI, 46–72) and 41% (95% CI, 21–64), respectively (Table 3 and Fig. 2). Specificity was 97–99% in all subgroups defined by the combination of BI-RADS density score and MTR marker.

**Table 3:** Sensitivity and specificity by the combination of density score (BI-RADS) and texture marker (MTR), Denmark 2012–2013

Measure	True positive, *N* (%)	False positive, *N* (%)	True negative, *N* (%)	False negative, *N* (%)	Sensitivity, % (95% CI)	Specificity, % (95% CI)
BI-RADS 1	88 (0.56)	204 (1.30)	15,327 (97.97)	25 (0.16)	78 (69–85)	99 (98–99)
MTR 1	52 (0.51)	138 (1.35)	10,054 (98.03)	12 (0.12)	81 (70–90)	99 (98–99)
MTR 2	27 (0.75)	38 (1.05)	3542 (97.90)	11 (0.30)	71 (54–85)	99 (99–99)
MTR 3	8 (0.56)	24 (1.68)	1397 (97.69)	1 (0.07)	82 (48–98)	98 (98–99)
MTR 4	1 (0.29)	4 (1.18)	334 (98.24)	1 (0.29)		
BI-RADS 2	187 (0.86)	613 (2.82)	20,903 (96.04)	61 (0.28)	75 (70–81)	97 (97–97)
MTR 1	32 (0.90)	100 (2.80)	3425 (95.97)	12 (0.34)	73 (57–85)	98 (97–98)
MTR 2	63 (0.80)	238 (3.01)	7592 (95.96)	19 (0.24)	77 (66–85)	97 (97–97)
MTR 3	63 (1.04)	162 (2.67)	5831 (96.03)	16 (0.26)	80 (64–88)	97 (97–98)
MTR 4	29(0.69)	113 (2.68)	4055 (96.30)	14 (0.33)	67 (51–81)	97 (97–98)
BI-RADS 3	127 (0.86)	420 (2.83)	14,222 (95.93)	57 (0.38)	69 (62–76)	97 (97–97)
MTR 1	2 (0.92)	4 (1.84)	209 (96.31)	2 (0.92)	71 (52–86)	97 (96–98)
MTR 2	20 (1.00)	60 (3.00)	1913 (95.65)	7 (0.35)		
MTR 3	55 (1.05)	150 (2.85)	5036 (95.80)	16 (0.30)	77 (66–87)	97 (97–98)
MTR 4	50 (0.68)	206 (2.80)	7064 (96.08)	32 (0.44)	61 (46–72)	97 (97–98)
BI-RADS 4	17 (0.62)	67 (2.42)	2660 (96.27)	19 (0.69)	47 (30–65)	98 (97–98)
MTR 1	0	0	54 (98.18)	1 (1.82)	57 (29–82)	98 (97–99)
MTR 2	1 (0.82)	2 (1.64)	119 (97.54)	0		
MTR 3	7 (0.79)	22 (2.50)	847 (96.14)	5 (0.57)		
MTR 4	9 (0.53)	43 (2.52)	1640 (96.19)	13 (0.76)	41 (21–64)	97 (97–98)

## Discussion

*Our study showed that the sensitivity of screening mammography could be further differentiated by combining density scoring with a texture marker*. On the one hand, the screening sensitivity was around 80% in women with a fatty and homogenous texture of their breast tissue. These women constituted about half of the screening population. On the other hand, screening sensitivity was down to 67% in women with a fatty breast but with a heterogenous texture (= BI-RADS density score 2 and MTR marker 4). These women constituted about 8% of the screening population. Among women with dense breasts, the sensitivity was also particularly low for women with a heterogenous texture: 61% for BI-RADS density score 3 and MTR marker 4, and 41% for BI-RADS density score 4 and MTR marker 4. Screening specificity was high in all subgroups resulting from the combination of density and texture levels.

The high correlation between density and texture was found also in a Dutch study, where density was assessed automatically using the Volpara Density (version 1.5.0, Volpara Health Technologies, Wellington, New Zealand) [16] and where texture was assessed with the same Biomediq MTR marker as used in the present study [13]. Nevertheless, adding the residuals (relative to density) of the texture marker to a model with density improved prediction of breast cancer risk in the Dutch data [14], and the improvement was larger for interval than for screen-detected cancers. This corresponds well to our findings, where stratification within BI-RADS density score categories using the MTR marker identified lower sensitivity subgroups.

In a UK study, volumetric density was assessed by the Volpare system, described above [16] and a texture scoring algorithm developed by the authors [17]. In the validating part of their study, the results indicated that their texture risk score for breast cancer added independent predictive information to the volumetric percent density, especially so for interval cancers, indicating that also in their study the combination of density and texture has the potential to improve screening sensitivity using, e.g., targeted supplemental imaging.

Comparisons of screening outcomes between the USA and Europe are difficult due to the variations in screening interval and definition of interval cancers. The US Breast Cancer Surveillance Consortium (BCSC) from 2002 to 2011 showed for women aged 50–59 years, the sensitivity vary from 89.6% for women with BI-RADS density score 1 to 71.3% for women with BI-RADS density score 4; the numbers for women aged 60–69 years were 92.5% and 65.5%, respectively [18]. The BCSC sensitivity data were thus both at a higher level and with a smaller range between BI-RADS density scores 1 and 4 than the Danish data. This was partly explained by the predominantly annual screening in the USA versus the biennial screening in Denmark, and consequently also by counting of interval cancers during 1 year versus 2 years since the last screen.

From studies where efforts have been made to standardize the data for comparison, screening in the USA is known to have a lower specificity than screening in Europe [18–20]. The BCSC data from 2002 to 2011 showed some variation in specificity across BI-RADS density score, for women aged 50–59 years being 94.7%, 90.6%, 88.2%, and 90.5%, for scores 1 to 4, respectively, and for women aged 60–69 years being 94.9%, 91.7%, 90.0%, and 92.5%, respectively [21]. In crude terms, 5 out of 100 women with BI-RADS density score 1, who turned out to be free of breast cancer, were recalled for extra examinations, while this was the case for 10 out of 100 for women with BI-RADS density scores 2–4. The equivalent numbers on the Danish data reported here were 1 out of 100 for women with BI-RADS density score 1 and 2–3 out of 100 for women with BI-RADS density scores 2–4. These differences in specificity explained in part the overall higher, and the more narrow range, of sensitivity scores across BI-RADS density scores in the BCSC than in the Danish data. The US women declared breast cancer-free at screening were simply more tightly sorted as to possible malignancy on their screening mammogram than the Danish women in this group, and this was in particular true for US women with dense breasts. It is on this basis understandable that the potential benefits of supplementary screening for women with dense breast, implied in the Breast Density Legislation “must be carefully weighed against the substantial risk of false-positive findings” [8], as 10 out of 100 women entitled to this supplementary screening would even without this experience a false-positive screen during their routine screening examination. The recently published small study by Mainprize et al. [22] indicated that the addition of various biometric and image-based parameters increased the predictability of nonscreen-detected cancers.

The strength of our study was that the mammography register included all mammograms taken in the target population in the study period and that follow-up for breast cancer and DCIS was complete. A limitation was that a radiology-assessed density measure was used, and we were not able to evaluate the possible impact of subjectivity and inter-observer variation [23]. The density and texture scores were not independent; for instance, among the 5% of screened women with extremely dense breasts, the majority, 62%, had the highest texture marker. The MTR score was developed using images from a Hologic Selenia FFDM system [13], while the images in our dataset came from a Siemens FFDM system. To what extent this could have influenced the results is not known.

## Conclusion

Our study showed on the one hand a high screening sensitivity in women with fatty homogenous breast tissue. These women constituted around half of the screening population. In the European context, where a double reading of screening mammograms is the standard, our finding might lead to considering single reading for these women. On the other hand, the combination of BI-RADS density score and MTR marker identified subgroups with low sensitivity with finer granularity than the BI-RADS density score alone indicating that this combination could help targeting resources for supplementary screening.

## Data Availability

The datasets generated and/or analyzed during the current study are not publicly available due to the General Data Protection Regulation but are available from the corresponding author on reasonable request and the permission from the Danish Data Inspection Agency.
